# Disrupted access and diverging pathways to sexual health services during COVID-19: insights from the PrEP Impact Trial in England

**DOI:** 10.1186/s12879-026-14044-5

**Published:** 2026-07-28

**Authors:** Dana Ogaz, Chiara Chiavenna, Holly D. Mitchell, Andrea Cartier, Rainer Golombek, Andre Charlett, Sajjida Jaffer, Victor Diamente, Hamish Mohammed, Elizabeth Carlin, Patricia Williams, Matt Phillips, O. Noel Gill, John Saunders, Ann K. Sullivan

**Affiliations:** 1 UK Health Security Agency, Blood Safety, Hepatitis, STI & HIV (BSHSH) Division, 61 Colindale Avenue, London, NW9 5EQ UK; 2 Chelsea and Westminster Hospital NHS Foundation Trust, London, UK; 3 The National Institute for Health and Care Research Health Protection Research Unit (NIHR HPRU) in Blood Borne and Sexually Transmitted, Infections at University College London, London, UK; 4 Sherwood Forest Hospitals NHS Foundation Trust, Sutton-in-Ashfield, UK; 5 Milton Keynes University Hospital NHS Foundation Trust, Milton Keynes, UK; 6 North Cumbria Integrated Care NHS Foundation Trust, Penrith, UK; 7 University College London, London, UK

**Keywords:** HIV PrEP, COVID-19, England, Sexual health services, STI, HIV

## Abstract

**Background:**

Using data from the HIV pre-exposure prophylaxis (PrEP) Impact trial, an implementation trial of PrEP across sexual health services to inform routine PrEP commissioning in England, we examined sexual health service use and PrEP need during UK COVID-19 social restrictions (March-November 2020) to assess the pandemic’s impact on PrEP delivery in England. This is the first analysis extending trial data beyond February 2020.

**Methods:**

We collated monthly aggregate data from 157 PrEP Impact Trial clinics in England (October 2017–November 2020), focusing on gay, bisexual, and other men who have sex with men (MSM). For trial participants and non-trial attendees (NTA), we examined trends in service use, STI/HIV test positivity, PrEP need, and prescriptions (30, 60, 90, ≥ 120 tablets). We also assessed changes in the enrolment patterns of key subgroups (aged 16–24, ethnic minority background, and bacterial STI history) over time. COVID-19 restrictions were defined as March–July and September–November 2020.

**Results:**

Attendances markedly decreased between February and April 2020 (-34% for trial participants; -78% for NTA). By July 2020, trial participant attendances had nearly returned to pre-lockdown levels, while NTA attendances remained more than 50% lower than in January. HIV and STI test positivity increased more among NTA than trial attendees during restrictions, reaching 0.95% [95% CI 0.51–1.63] and 18.9% [95% CI 17.0-20.9] respectively. This suggests triage towards higher risk individuals but also highlights likely unmet need among others. The proportion of clinic attendees with PrEP need also increased during the pandemic, and short-lived increases to longer term prescriptions were observed. Demographic shifts during restrictions showed decreased in-person enrolment among younger people and increased representation of ethnic minority groups.

**Conclusions:**

Structured PrEP delivery preserved access among trial participants during COVID-19-related disruptions, but non-trial attendees, despite persistent risk, faced greater barriers and slower recovery. These disparities underscore the need for resilient, flexible delivery models that can sustain HIV prevention in periods of major service disruptions. With continued PrEP scale-up, addressing equity gaps for those outside established care pathways will be essential to prevent widening disparities and ensure the success of HIV prevention efforts.

**Trial registration:**

The study is registered with ClinicalTrials.gov (Prospective, non-interventional trial NCT03253757; registered 06/16/2017).

**Supplementary Information:**

The online version contains supplementary material available at https://doi.org/10.1186/s12879-026-14044-5.

## Background

The COVID-19 pandemic and associated restrictions led to major disruptions to healthcare access and provision. The UK government introduced measures such as social distancing and travel restrictions to minimise COVID-19 transmission from March 2020. The first of three national lockdowns lasted from 23 March to 4 July 2020 [1]. New social gathering restrictions and local lockdown continued from September 2020 onwards, and a second national lockdown came into effect on 5 November 2020. These measures severely affected UK healthcare services, including sexual health services (SHS), disrupting the provision of STI and HIV prevention interventions not provided remotely (e.g., vaccination) [2]. Disruptions to sexual health services were reported internationally [3], including in high-income countries with established HIV pre-exposure prophylaxis (hereafter, PrEP) programmes [4–6].

From October 2017 to March 2020, PrEP in England was primarily provided through 157 sexual health services participating in the PrEP Impact Trial [7], an implementation study to inform routine PrEP commissioning. Participants clinically assessed as eligible for PrEP and agreeing to quarterly follow-up visits (face-to-face or online) were enrolled at trial clinics and received PrEP prescriptions for up to 6-months [7–9]. During England’s social restrictions, the PrEP Impact trial continued enrolling new participants until planned enrolment end (July 2020) and continued follow-up visits, including PrEP provision, through the trial conclusion in November 2020. While many SHS remained open for complex cases, those using PrEP and high priority groups [10–12], face-to-face services were largely reduced due to staffing redeployment [11]. During periods of social restrictions, all SHS users were subject to online or telephone triage before attending services. Consequently, overall STI and HIV testing volumes fell substantially during lockdown periods, indicating reduced access to routine (including asymptomatic) screening, although laboratory testing itself was not formally suspended. Through restrictions, the PrEP Impact Trial remained open to recruitment and follow-up, with protocol amendments that included dispense of 6-monthly prescriptions with follow-up assessment through e-services (hereafter online services) (i.e. STI self-sampling activity captured as online service activity [13]) and remote appointments. Outside of the trial, PrEP was also available through online and private sourcing but with STI, HIV and renal monitoring provided through publicly funded SHS. Routine commissioning of PrEP in England, where PrEP became nationally available across sexual health services through the National Health Service (NHS), began from October 2020 [14].

To minimise the impact of COVID-19 restrictions on key PrEP Impact Trial outcomes (e.g. PrEP eligibility and need, STI and HIV testing), the study was shortened to include data up to 29 February 2020 [7]. This is the first report extending PrEP Impact trial data through COVID-19 social restrictions to the trial endpoint in November 2020. We descriptively examine SHS use, STI/HIV testing and diagnoses, PrEP need and prescriptions, and enrolee profiles during UK social restrictions to assess the pandemic’s impact on PrEP delivery and service use.

## Methods

### Study methods and participants

Using trial data from 157 sexual health clinics participating in the PrEP Impact trial in England, we present aggregate monthly totals from February 2020 (the period of interest for main trial analyses [7]) to November 2020 (end of trial data collection), unless otherwise specified, for individual outcomes described below. Analyses were restricted to gay, bisexual, and other men who have sex with men (MSM), the primary trial recruitment group. This population represented the highest HIV risk group in the UK and accounted for the majority of PrEP users.

Full trial details have been previously described [7]. A summary of trial participants (people enrolled) and non-trial attendees (NTA; people never enrolled) is found in Appendix I (MSM participant flow chart). Briefly, trial participants included HIV-negative individuals aged ≥ 16 who were clinically assessed as eligible for PrEP attending participating SHS between 13 October 2017 and 12 July 2020. NTA included HIV-negative individuals aged ≥ 16 attending the same clinics during the study period. Trial data, consisting of electronic case report forms (eCRFs) of PrEP prescription at clinic visits, were linked with informed consent to national STI surveillance (GUMCAD STI Surveillance System) [9].

For the present analysis, COVID-19 social restriction periods were defined as March to July 2020 (first national lockdown) and September to November 2020 (local lockdowns prior to second national lockdown).

### Service use in trial clinics, October 2017 to November 2020

Service use trends were examined by calculating monthly totals of trial enrolees, trial participants and attendances; for NTAs, numbers of attendees and attendances from trial start to end (October 2017-November 2020). The percentage of clinic attendances by trial participants (enrolment and follow-up visits) was also calculated (participant attendances/ all attendances x 100%). Due to limitations in data reporting and data governance restrictions, online service activity was underrepresented and not added to NTA records. However, for trial participants online service use was integrated in attendance records, though this only represented < 1% of all attendances.

### STI/HIV tests, diagnoses, and positivity, January 2019 to November 2020

Monthly number of individual tests and diagnoses were collated for trial participants and NTA to examine trends from January 2019 to November 2020. As per methodology used for STI incidence calculations in main trial analysis [7], prevalent STI diagnoses, or diagnoses made at trial participant enrolment or NTA first attendance, were excluded from monthly totals. STI test positivity was computed as number of individual STI diagnoses/number of individual STI tests x 100%, and HIV test positivity as number of HIV diagnoses/number of HIV tests x 100%, with 95% confidence intervals (CI).

### PrEP need, January 2019 to November 2020

NTAs at risk of HIV acquisition were identified as per previous trial methods [7] (i.e. attendees meeting at least one risk criterium among reporting a rectal bacterial STI diagnosis, use of HIV post-exposure prophylaxis (PEP), being a sex worker, being a sexual contact of someone diagnosed with HIV or syphilis, and/or having had two or more HIV tests in the previous year).

We examined monthly PrEP-need by combining the number of trial participants, NTA considered at risk of HIV acquisition (described above), HIV diagnoses in trial participants (all) and NTA not considered at risk of HIV acquisition, and those ever obtaining PrEP from another source (i.e., not trial sourced). For monthly totals, those who ever reported obtaining PrEP from another source were counted this way throughout the analysis period. Trends of PrEP need were computed as the percentage of clinic attendees with PrEP need (PrEP need/all trial clinic attendees x 100%), and the proportion of clinic attendees with PrEP need met by the Impact trial as trial participants/total PrEP need x 100%, with 95% CIs.

### PrEP prescriptions, January 2019 to November 2020

To examine changes in PrEP prescriptions, we collated the number and type of monthly prescriptions (enrolment or follow-up). We present monthly totals (30, 60, 90, ≥ 120 tablets), where specified. Data underreporting limited examinations of PrEP regimen switching (e.g. between daily and event-driven use) through the trial period.

### Enrolee profiles by period, October 2017 to November 2020

Shifts in enrolment profiles were assessed by calculating the percentages of trial enrolees aged 16–24, of those from an ethnic minority group (i.e. reporting Black, Asian, Mixed or other ethnicity), and of those with a bacterial STI history (chlamydia, gonorrhoea, or syphilis) one year prior to enrolment by approximately 6-monthly periods from the start of trial enrolment (October 2017) to enrolment end (July 2020). NTA characteristics are similarly assessed based on first attendance during the period of interest.

Time periods used to assess enrolee profiles were Period 1 (October 2017-March 2018), Period 2 (April 2018-September 2018), Period 3 (October 2018-March 2019), Period 4 (April 2019-September 2019), Period 5 (October 2019-February 2020), and Period 6 (March 2020-July 2020). These intervals were pragmatically set, with Periods 5 and 6 shortened to capture pre- and post- COVID-19 restrictions respectively.

## Results

### Service use in participating trial clinics, October 2017 to November 2020

From October 2017 to July 2020, 23,178 MSM participants enrolled in the PrEP Impact trial (24,215 total trial participants, not shown). Enrolment continued during COVID-19 restrictions, with 12% of MSM trial participants recruited during the first lockdown period (March–July 2020). Between March and November 2020, there were 119,518 attendances across trial clinics (58,043 by trial participants and 61,475 by NTA).

**Fig. 1:**
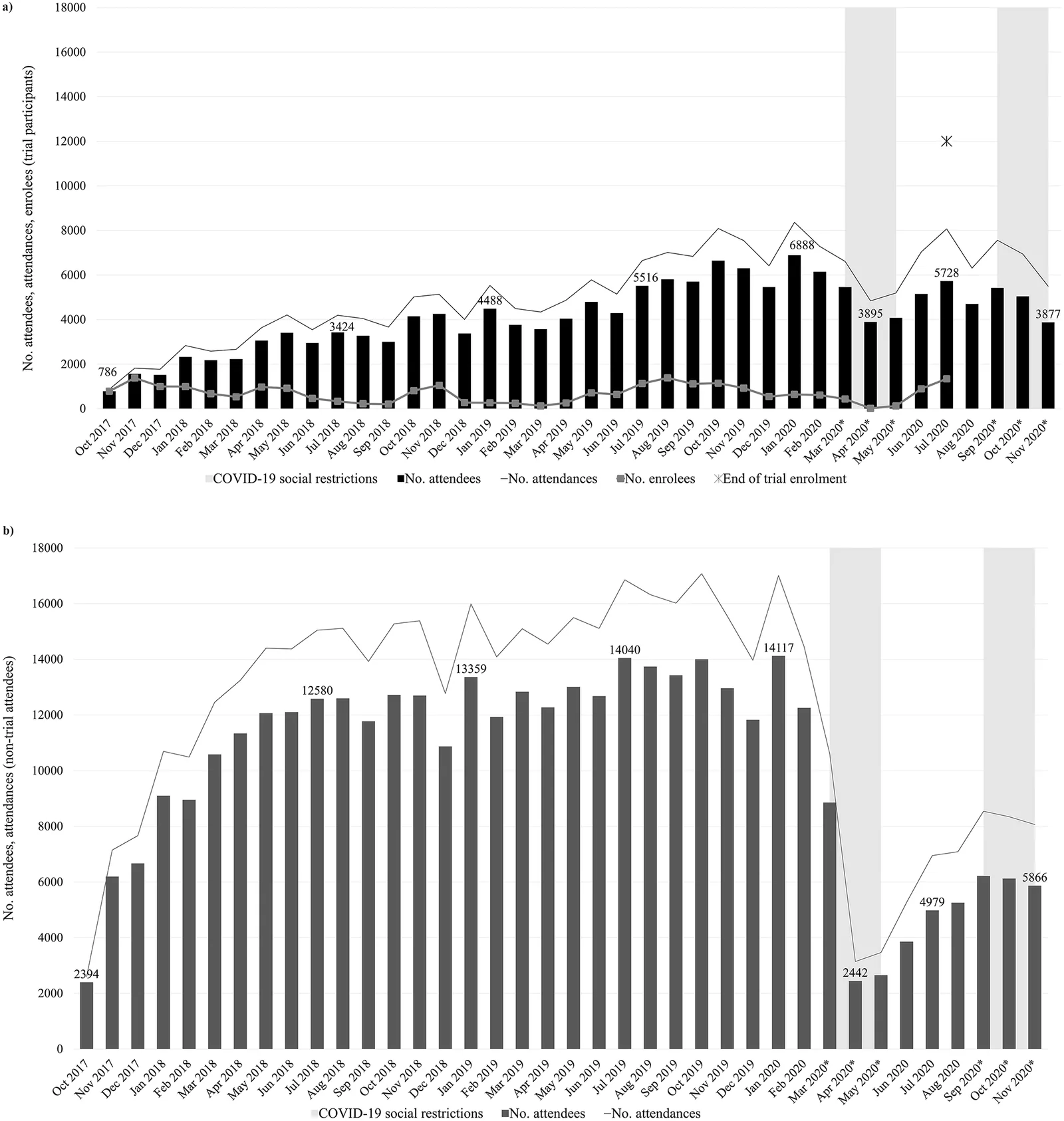
Service use trends in trial participants and non-trial attendees to clinics participating in the PrEP Impact trial, October 2017-November 2020. **a**) Trial participant. **b**) Non-trial attendees. *COVID-19 social restrictions (March-May 2020, September-Nov 2020). Note scale

Through the study period, trial enrolment fluctuated (Fig. 1a, Appendix II), peaking at 1,383 enrolees in August 2019 and dropping to 20 during April 2020’s national lockdown, before rebounding to 1344 by July 2020. Attendance trends mirrored enrolment (Fig. 1a, Appendix II Number of enrolees, attendees and attendances among trial participants and non-trial attendees to clinics participating in the PrEP Impact trial, October 2017-November 2020), with trial participants attendances dropping by 42% from January (8,363) to April 2020 (4,838) and NTA attendances falling by 82% (17,007 to 3,142 attendances) (Fig. 1b, Appendix II). Trial participant attendances recovered to 8,069 by July 2020, while NTA attendances remained below half of January levels (6,949). Despite this recovery in trial participants following the first lockdown period, attendances declined again during subsequent lockdown periods (September 2020-November 2020) alongside gradual, but consistent attendance recovery among non-trial attendees.

The proportion of attendances by trial participants increased up to the first lockdown (25% in October 2017 to 34% in February 2020). With decreases in overall attendance numbers, trial participants comprised over 61% of all clinic attendance activity in April 2020, decreasing to 41% through November 2020 as attendance numbers began to recover (Appendix II).

### STI/HIV tests, diagnoses, and test positivity, January 2019 to November 2020

**Fig. 2:**
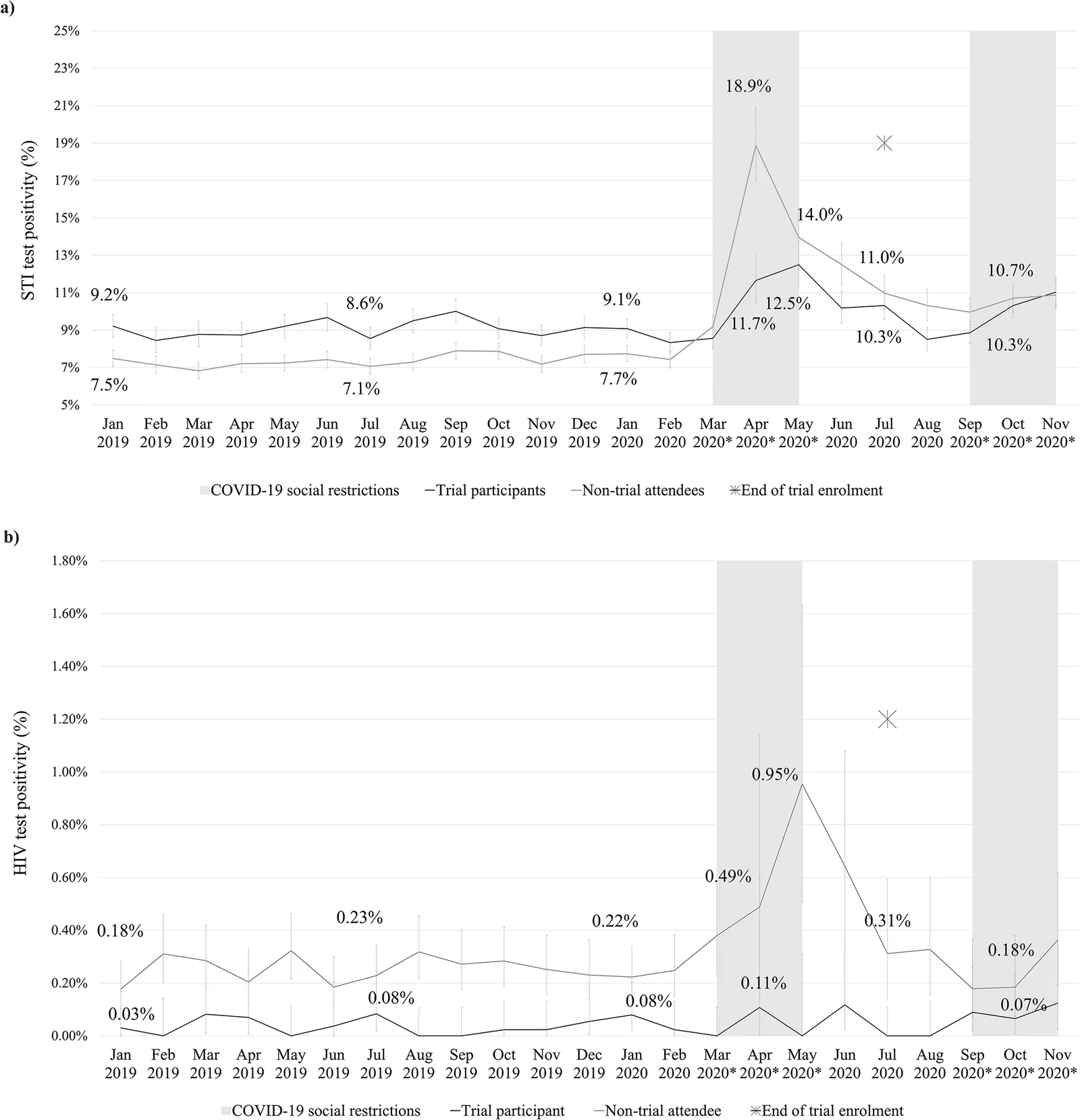
STI and HIV test positivity in trial participants and non-trial attendees to clinics participating in the PrEP Impact trial, January 2019-November 2020. **a**) STI test positivity. **b**) HIV test positivity. *COVID-19 social restrictions (March-May 2020, September-Nov 2020). Note scale

From January 2019 to February 2020, STI test positivity was higher in trial participants (8–9%) than in NTA (< 8%) (Fig. 2a). During lockdowns, STI positivity shifted, peaking at 18.9% (95% CI 17.0–20.9) in NTA in April 2020 versus 11.7% (95% CI 10.4–13.0) in trial participants (Appendix II). Full STI testing information is found in Appendix III (Number of STI tests, diagnoses, and test positivity among trial participants and non-trial attendees to clinics participating in the PrEP Impact trial, January 2019-November 2020).

In contrast, HIV test positivity was consistently higher in NTA than trial participants, peaking in May 2020 at 0.95% (95% CI 0.51–1.63) among NTA and 0.12% (95% CI 0.03–0.36) in trial participants by November 2020 (Fig. 2b, Appendix IV Number of HIV tests, diagnoses, and test positivity among trial participants and non-trial attendees of clinics participating in the PrEP Impact trial, January 2019-November 2020). Full HIV testing information is found in Appendix IV.

### PrEP need, January 2019 to November 2020

**Fig. 3:**
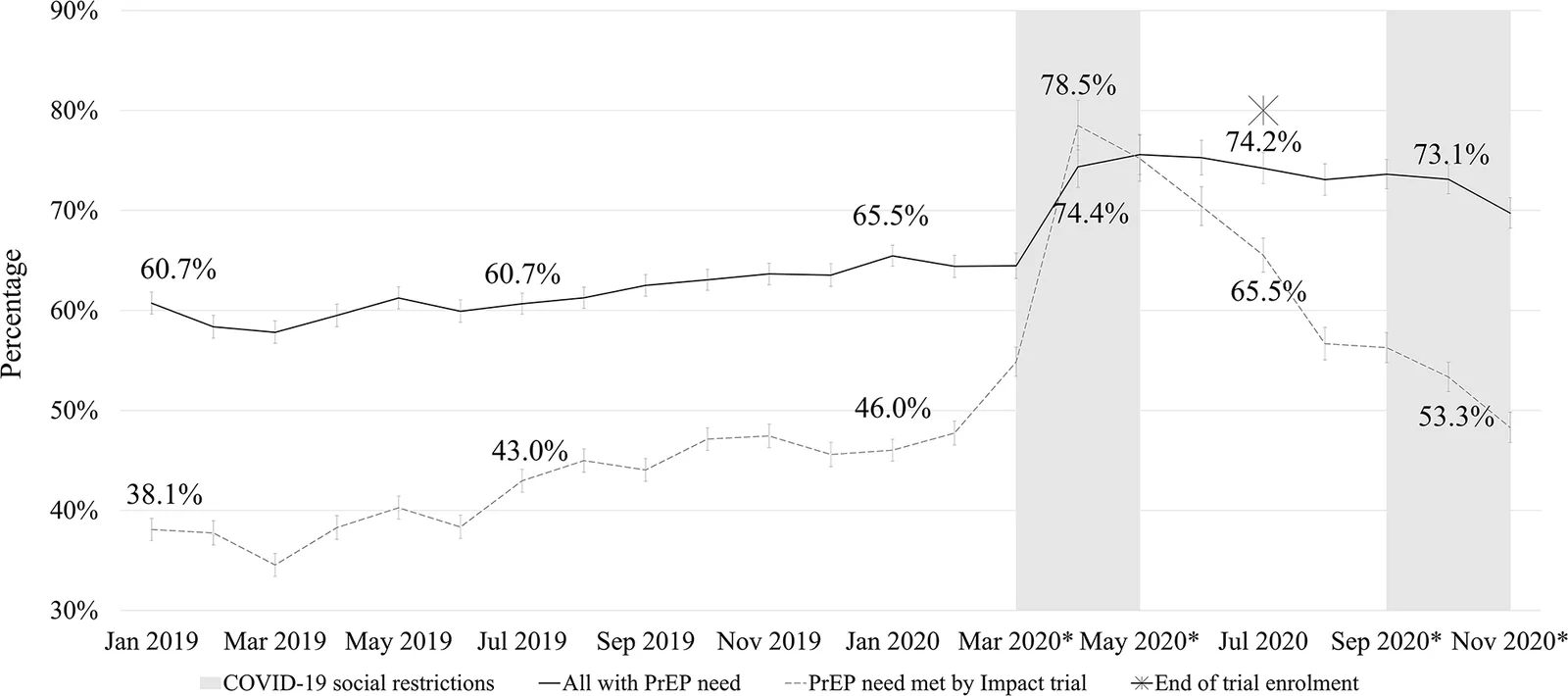
Percentage of clinic attendees with PrEP need and percentage of attendees with PrEP need who had need met by the PrEP Impact trial, January 2019-November 2020. *COVID-19 social restrictions (March-May 2020, September-Nov 2020). Note scale

Levels of PrEP need gradually increased from 61% (95% CI 60–62) in January 2019 to 64% (95% CI 63–66) in February 2020 (Fig. 3, Appendix V Number of attendees with PrEP need and percentage of attendees with PrEP need who had need met by the PrEP Impact trial, January 2019-November 2020). During the lockdown period, PrEP need increased to 76% (95% CI 74–78) in May 2020, and then decreased through the end of data collection. PrEP need met by the PrEP Impact trial steadily rose through the study period, peaking in April 2020 (79% [95% CI 76–81]), when trial participants comprised the highest percentage of clinic attendances (Appendix II). PrEP need met by the Impact trial fell during the first lockdown period through to the trial conclusion. Full PrEP need information is found in Appendix V.

### PrEP prescriptions, January 2019 to November 2020

Over 82,000 PrEP prescriptions were issued during the study period; most, where specified, were for 90 tablets. During the first lockdown, prescriptions of ≥ 120 tablets peaked at 1,632 in May 2020 (57% of 2,856 prescriptions) before shifting back to 90 tablets from June 2020 onwards (Table 1).

**Table 1:** Number and percentage of PrEP prescriptions and prescriptions type, January 2019-November 2020

	No. SHS attendees	No. PrEP prescriptions***	PrEP prescription type			
			30 tablets *n* (row %)	60 tablets *n* (row %)	90 tablets *n* (row %)	≥ 120 tablets *n* (row %)
Jan 2019	4,488	3,334	80 (2%)	107 (3%)	3,143 (94%)	4 (< 1%)
Feb 2019	3,762	2,657	71 (3%)	88 (3%)	2,494 (94%)	4 (< 1%)
Mar 2019	3,570	2,392	80 (3%)	69 (3%)	2,240 (94%)	3 (< 1%)
Apr 2019	4,040	2,939	99 (3%)	102 (3%)	2,737 (93%)	1 (< 1%)
May 2019	4,794	3,590	145 (4%)	136 (4%)	3,307 (92%)	2 (< 1%)
Jun 2019	4,291	2,982	112 (4%)	141 (5%)	2,724 (91%)	5 (< 1%)
Jul 2019	5,516	4,185	136 (3%)	179 (4%)	3,865 (92%)	5 (< 1%)
Aug 2019	5,805	4,350	171 (4%)	177 (4%)	3,998 (92%)	4 (< 1%)
Sep 2019	5,700	4,094	224 (5%)	197 (5%)	3,667 (90%)	6 (< 1%)
Oct 2019	6,643	4,876	117 (2%)	244 (5%)	4,507 (92%)	8 (< 1%)
Nov 2019	6,301	4,628	132 (3%)	195 (4%)	4,297 (93%)	4 (< 1%)
Dec 2019	5,457	3,868	132 (3%)	169 (4%)	3,559 (92%)	8 (< 1%)
Jan 2020	6,888	5,076	189 (4%)	193 (4%)	4,654 (92%)	40 (1%)
Feb 2020	6,144	4,542	123 (3%)	154 (3%)	3,897 (86%)	368 (8%)
Mar 2020*	5,458	3,995	77 (2%)	126 (3%)	2,848 (71%)	944 (24%)
Apr 2020*	3,895	2,597	33 (1%)	68 (3%)	882 (34%)	1,614 (62%)
May 2020*	4,077	2,856	42 (1%)	83 (3%)	1,099 (38%)	1,632 (57%)
Jun 2020	5,149	3,505	64 (2%)	84 (2%)	3,310 (94%)	47 (1%)
Jul 2020**	5,728	3,907	126 (3%)	138 (4%)	3,637 (93%)	6 (< 1%)
Aug 2020	4,701	2,667	147 (6%)	156 (6%)	2,354 (88%)	10 (< 1%)
Sep 2020*	5,422	3,609	154 (4%)	150 (4%)	3,295 (91%)	10 (< 1%)
Oct 2020*	5,043	3,219	96 (3%)	99 (3%)	2,987 (93%)	37 (1%)
Nov 2020*	3,877	2,166	47 (2%)	46 (2%)	2,035 (94%)	38 (2%)

*COVID-19 social restrictions (March-May 2020, September-Nov 2020).**End of trial enrolment. ***Where specified

Enrolee profiles by period, October 2017 to November 2020.

Between Periods 1 and 5, the proportion of trial enrolees aged 16–24 increased (10.3% to 18.3%), while representation from ethnic minority groups (Black, Asian, Mixed, or other ethnic groups) fluctuated slightly (17.2% to 17.9%). Proportional representation of younger ages and ethnic minority groups was higher across non-trial attendees but did not show variations through Periods 1–5 (Table 2). During Period 6, affected by COVID-19 restrictions, younger enrolees dropped to 14.4%, and ethnic minority representation rose to 21.7%. Bacterial STI history among trial enrolees decreased from 43.9% in Period 1 to 37.7% in Period 6, while increasing in NTA from 24.7% to 30.2% over the same periods (see Table 2).

**Table 2:** Number of trial enrolees and non-trial attendees by select age, ethnicity, and markers of risk by period, October 2017-July 2020

	a) Aged 16–24		b) Ethnic minority group		c) Bacterial STI in the last year	
	Trial participants*	Non-trial attendees**	Trial participants*	Non-trial attendees**	Trial participants*	Non-trial attendees**
Period 1 (Oct 2017-Mar 2018)	554 (10.3)	7,247 (21.6)	862 (17.2)	6,272 (20.0)	2,355 (43.9)	8,290 (24.7)
Period 2 (Apr 2018-Sep 2018)	457 (14.7)	11,760 (22.9)	484 (16.4)	8,718 (18.4)	1,310 (42.1)	12,253 (23.9)
Period 3 (Oct 2018-Mar 2019)	422 (15.2)	12,064 (23.8)	523 (20.3)	8,928 (18.9)	1,168 (42.1)	12,235 (24.1)
Period 4 (Apr 2019-Sep 2019)	786 (15.0)	12,394 (23.0)	913 (18.8)	9,436 (18.8)	2,145 (40.9)	13,024 (24.1)
Period 5 (Oct 2019-Feb 2020)	710 (18.3)	10,280 (22.1)	628 (17.9)	8,255 (19.1)	1,518 (39.2)	11,910 (25.7)
Period 6 (Mar 2020-Jul 2020)***	406 (14.4)	4,164 (22.3)	554 (21.7)	3,376 (19.4)	1,061 (37.7)	5,623 (30.2)
All	3,335 (14.4)	57,909 (22.7)	3,964 (18.5)	44,985 (19.0)	9,557 (41.2)	63,335 (24.9)

*Trial participants enrolled into trial during period of interest. ** Non-trial attendee during period of interest. ***COVID-19 social restrictions (March-May 2020, September-Nov 2020)

## Discussion

This study offers one of the most comprehensive views to date on how COVID-19-related restrictions affected SHS use and PrEP delivery in England. Using data from the PrEP Impact Trial, we demonstrate how embedded PrEP service models maintained continuity of care for many users despite national service disruptions. The pandemic exposed vulnerabilities in routine SHS access but also revealed resilience within structured delivery mechanisms like trial-based care. These findings offer critical insight into how service prioritisation, constrained capacity, and changing care-seeking patterns shaped access and health outcomes during a public health emergency that had profound impacts on sexual health service delivery.

Internationally, studies have documented PrEP discontinuation, interruptions in HIV testing, and a shift from daily to event-based dosing in the early stages of the pandemic [15, 16]. Such changes could be attributed to both service closures and shifts in sexual behaviour. In contrast, UK-based behavioural surveys report less pronounced behavioural change among MSM and more stable PrEP continuation than observed elsewhere [17–19]. Our findings align with this, showing only a modest decline in clinic attendances among trial participants during restrictions, suggesting continued engagement in care. While we did not measure behaviour directly, better service continuity among trial participants, compared to the sharper declines seen in non-trial attendees (NTA), likely reflects sustained access to PrEP and related prevention services among those already linked to care. However, as behavioural changes were not directly measured, we cannot exclude differential use of other HIV prevention strategies among trial participants and NTA (e.g., reduced sexual activity or increased condom use) during lockdown periods. Alongside, differences between trial participants and non-trial attendees may partly reflect underlying differences in engagement with healthcare services rather than the effect of the trial alone.

In contrast, NTA experienced more pronounced service interruptions and slower returns to care. Elevated HIV and STI test positivity among NTA during restrictions likely reflected a shift in the composition of attendees, as reduced face-to-face access and triage before attendance may have concentrated service use among individuals with symptoms, known exposure, recent STI history, or more urgent perceived need. These disparities suggest that individuals outside structured PrEP pathways were less consistently able to access services when availability was limited. The inverse trends in STI history between trial participants and NTA may further reflect diverging pathways into care, with stable retention among engaged users versus risk-driven entry among those not formally linked to services. At the same time, it remains plausible that other service users at risk were missed. This interpretation is supported by survey findings indicating continued sexual activity during social restrictions alongside unmet sexual health needs [17, 18, 20, 21], and by national surveillance showing a post-lockdown rise in STI diagnoses [22, 23], likely reflecting deferred care and accumulated unmet need among those who did not access services during the height of the pandemic.

The marked increase in ≥ 120-tablet prescriptions during the first lockdown is consistent with temporary multi-month dispensing to maintain PrEP continuity, potentially reducing dispensing need as services reopened.

### Limitations

This analysis is limited by the lack of available online service data for NTA, which prevents a full accounting of how online service delivery contributed to continuity of care. National surveillance data indicate that online testing represented a majority of STI/HIV testing by May 2020 [2], suggesting a key component of care was underrepresented. Trial participants’ attendance records included integrated online service visits, but these comprised a small fraction of all attendances, likely underestimating the role of remote modalities. As online sexual health service activity was not fully captured for non-trial attendees, this may have led to an underestimation of service use and testing during lockdown periods. Therefore, we can only speculate that the decline in younger enrolees during restrictions may reflect a preference for, or greater ability to access, online services, which were not captured in trial data. In contrast, the rise in ethnic minority representation among in-person enrolees may reflect differential access to, uptake of, or suitability of online modalities across population groups. This interpretation is consistent with evidence from England showing that online postal self-sampling (OPSS) uptake varies by sociodemographic characteristics, with larger relative increases among White British/Irish users and people living in less deprived areas, and with recommendations to maintain clinic-based testing for those unable or unwilling to access testing online [25]. Other countries with robust online service platforms may be better positioned to evaluate online PrEP delivery models.

Our proxy for PrEP need should be interpreted cautiously, as it relied on sexual health service access and engagement that was substantially affected by the COVID-19 pandemic. Changes in service access, healthcare-seeking behaviour, and testing patterns may have affected the identification of individuals eligible for PrEP, meaning that observed trends may partly reflect changes in surveillance and service use rather than genuine changes in underlying PrEP need.

Finally, we performed primarily descriptive analyses; formal interrupted time-series or regression modelling was not performed, limiting our ability to quantify the independent effect of COVID-19 restrictions on observed trends.

### Implications

Our findings highlight the importance of adaptable, equitable PrEP delivery models as health systems prepare for future disruptions. The transition to nationally commissioned PrEP in England may be reducing the disparity between trial and non-trial users observed here. However, similar challenges persist in many other settings, especially where PrEP is not yet fully integrated into routine care.

Service adaptations during COVID-19, including remote follow-up, multi-month dispensing, and targeted triage, can inform the future of PrEP delivery. Long-acting PrEP modalities [24] and decentralised models may further buffer care from future shocks. However, these innovations must be deployed equitably, ensuring that those most at risk are not sidelined by digital divides, stigma, or triaging algorithms.

Interpreted through the Big Events framework, COVID-19 can be understood as one example of a broader class of structural disruptions that reshape service environments, health risks, and access to care. The differential continuity observed between trial participants and NTA highlights the need for resilient PrEP and STI service models that can maintain access during future disruptions, including climate-related emergencies, conflict, economic crises, or health-system shocks [26, 27].

## Conclusions

The COVID-19 pandemic disrupted sexual health services worldwide. In England, PrEP delivery through the Impact trial proved relatively resilient, especially for those already engaged in care. But for others, particularly those outside trial structures, access was more fragile. Planning for future PrEP programmes should prioritise flexible, inclusive delivery models that ensure prevention remains accessible even when services are under pressure.

## Supplementary Information

Below is the link to the electronic supplementary material.

Supplementary Material 1

## Data Availability

Requests for aggregate data collected for the study are reviewed by the Trial Steering Committee and UK Health Security Agency Impact Trial Data Sharing Review Panel in accordance with the HIV PrEP Impact Trial: PHE Data Handling Plan (February 2020) and a signed data sharing agreement. The study protocol and statistical analysis plan are also available upon request. Requests should be made via email to john.saunders@ukhsa.gov.uk.

## Declarations

### The Impact Study Group is composed of:

Monica Desai^1^ (monica.desai@ukhsa.gov.uk) Kaveh Manavi^2^ (Kaveh.Manavi@uhb.nhs.uk), Cecilia Priestley^3^ (Cecilia.Priestley@dchft.nhs.uk), Laura J Waters^4^ (lwaters@nhs.net), Ana Milinkovic^5^ (ana.milinkovic@nhs.net), Alan McOwan^5^ (alan.mcowan1@nhs.net), Claudia Estcourt^6^ (Claudia.Estcourt@gcu.ac.uk), Caroline A Sabin^7,8^ (c.sabin@ucl.ac.uk), Alison Rodger^7^ (alison.rodger@ucl.ac.uk), Deborah Gold^9^ (deborah.gold@nat.org.uk), Brian G Gazzard^5^ (brian.gazzard@nhs.net), Sheena McCormack^5,7^ (Sheena.McCormack@chelwest.nhs.uk), Yusef Azad^9^ (yusef.azad@nat.org.uk), Pallavi Chhibbar^5^ (Pallavi.Chhibbar@chelwest.nhs.uk), Flavien Coukan^1^ (flavien.coukan15@imperial.ac.uk), Matthew Hibbert^1^ (matthew.hibbert@ukhsa.gov.uk), Emily L Mason^1^ (emily.l.mason@ukhsa.gov.uk), Anthony Nardone^1^ (a.nardone@epiconcept.fr), Ola Oladela^5^ (Ola.Oladele@chelwest.nhs.uk), Roeann Osman^1^ (Roeann.Osman@ukhsa.gov.uk), Branca Pereira^5^ (branca.x.pereira@viivhealthcare.com), Hannah Reaney^5^ (Hannah.Reaney@ststcr.com), Beverley White-Alao^5^ (beverley.white-alao@ststcr.com).

^1^ UK Health Security Agency, London, United Kingdom (Monica Desai, Flavien Coukan, Matthew Hibbert, Emily Mason, Anthony Nardone, Roeann Osman).

^2^ University Hospitals Birmingham NHS Foundation Trust, Birmingham, United Kingdom (Kaveh Manavi).

^3^ Dorset County Hospital NHS Foundation Trust, Dorset, United Kingdom (Cecilia Priestley).

^4^ Central and North West London NHS Foundation Trust, London, United Kingdom (Laura J Waters).

^5^ Chelsea and Westminster Hospital NHS Foundation Trust, London, United Kingdom (Ana Milinkovic, Alan McOwan, Brian G Gazzard, Sheena McCormack, Pallavi Chhibbar, Branca Pereira, Hannah Reaney, Beverley White-Alao).

^6^ Glasgow Caledonian University, Glasgow, United Kingdom (Claudia Estcourt).

^7^ University College London, London, United Kingdom (Caroline A Sabin, Alison Rodger, Sheena McCormack).

^8^ The National Institute for Health and Care Research Health Protection Research Unit (NIHR HPRU) in Blood Borne and Sexually Transmitted Infections at University College London, London, United Kingdom (Caroline A Sabin).

^9^ National AIDS Trust, London, United Kingdom (Deborah Gold, Yusef Azad).

## Abbreviations

COVID-19: Coronavirus Disease 2019
SHS: Sexual health service
HIV: Human immunodeficiency virus
NHS: National Health Service
NTA: Non-trial attendees
MSM: Men who have sex with men
PrEP: HIV pre-exposure prophylaxis
STI: Sexually transmitted infection
UK: United Kingdom
