# Supplementary material for: Disrupted access and diverging pathways to sexual health services during COVID-19: insights from the PrEP Impact Trial in England
Source: BMC Infect Dis. 2026 Jul 28;26:1885. doi: 10.1186/s12879-026-14044-5 (PMC13711870; doi:10.1186/s12879-026-14044-5)
Supplement: Supplementary file 1 [file 12879_2026_14044_MOESM1_ESM.docx]

**Appendices**

Appendix I. MSM participant flow chart

Appendix II. Number of enrolees, attendees and attendances among trial participants and non-trial attendees to clinics participating in the PrEP Impact trial, October 2017-November 2020

Appendix III. Number of STI tests, diagnoses, and test positivity among trial participants and non-trial attendees to clinics participating in the PrEP Impact trial, January 2019-November 2020

Appendix IV. Number of HIV tests, diagnoses, and test positivity among trial participants and non-trial attendees of clinics participating in the PrEP Impact trial, January 2019-November 2020

Appendix V. Number of attendees with PrEP need and percentage of attendees with PrEP need who had need met by the PrEP Impact trial, January 2019-November 2020

**Appendix I. MSM participant flow chart**

**Appendix II. Number of enrolees, attendees and attendances among trial participants and non-trial attendees to clinics participating in the PrEP Impact trial, October 2017-November 2020**

|  | Trial participants (MSM) | | |  | Non-trial participants (MSM) | |  | Percentage of all attendances by trial participants** |  |
| --- | --- | --- | --- | --- | --- | --- | --- | --- | --- |
|  | No. enrolees | No. attendees | No. attendances |  | No. attendees | No. attendances |  |  |  |
| Oct 2017 | 786 | 786 | 872 |  | 2,394 | 2,597 |  | 25% |  |
| Nov 2017 | 1,380 | 1,570 | 1,819 |  | 6,191 | 7,147 |  | 20% |  |
| Dec 2017 | 998 | 1,518 | 1,771 |  | 6,666 | 7,666 |  | 19% |  |
| Jan 2018 | 995 | 2,327 | 2,835 |  | 9,095 | 10,692 |  | 21% |  |
| Feb 2018 | 671 | 2,175 | 2,580 |  | 8,949 | 10,488 |  | 20% |  |
| Mar 2018 | 532 | 2,231 | 2,666 |  | 10,580 | 12,459 |  | 18% |  |
| Apr 2018 | 972 | 3,057 | 3,634 |  | 11,333 | 13,245 |  | 22% |  |
| May 2018 | 918 | 3,407 | 4,208 |  | 12,061 | 14,399 |  | 23% |  |
| Jun 2018 | 462 | 2,948 | 3,547 |  | 12,096 | 14,372 |  | 20% |  |
| Jul 2018 | 333 | 3,424 | 4,191 |  | 12,580 | 15,044 |  | 22% |  |
| Aug 2018 | 223 | 3,274 | 4,046 |  | 12,593 | 15,113 |  | 21% |  |
| Sep 2018 | 206 | 3,002 | 3,661 |  | 11,769 | 13,925 |  | 21% |  |
| Oct 2018 | 809 | 4,145 | 5,017 |  | 12,718 | 15,275 |  | 25% |  |
| Nov 2018 | 1,049 | 4,255 | 5,133 |  | 12,695 | 15,382 |  | 25% |  |
| Dec 2018 | 277 | 3,378 | 4,004 |  | 10,868 | 12,770 |  | 24% |  |
| Jan 2019 | 266 | 4,488 | 5,530 |  | 13,359 | 15,987 |  | 26% |  |
| Feb 2019 | 246 | 3,762 | 4,493 |  | 11,928 | 14,085 |  | 24% |  |
| Mar 2019 | 126 | 3,570 | 4,341 |  | 12,830 | 15,096 |  | 22% |  |
| Apr 2019 | 260 | 4,040 | 4,868 |  | 12,267 | 14,546 |  | 25% |  |
| May 2019 | 713 | 4,794 | 5,779 |  | 13,007 | 15,496 |  | 27% |  |
| Jun 2019 | 641 | 4,291 | 5,144 |  | 12,671 | 15,110 |  | 25% |  |
| Jul 2019 | 1,130 | 5,516 | 6,646 |  | 14,040 | 16,856 |  | 28% |  |
| Aug 2019 | 1,383 | 5,805 | 7,011 |  | 13,736 | 16,320 |  | 30% |  |
| Sep 2019 | 1,116 | 5,700 | 6,834 |  | 13,424 | 16,018 |  | 30% |  |
| Oct 2019 | 1,144 | 6,643 | 8,088 |  | 13,998 | 17,076 |  | 32% |  |
| Nov 2019 | 925 | 6,301 | 7,546 |  | 12,957 | 15,555 |  | 33% |  |
| Dec 2019 | 545 | 5,457 | 6,411 |  | 11,820 | 13,963 |  | 31% |  |
| Jan 2020 | 644 | 6,888 | 8,363 |  | 14,117 | 17,007 |  | 33% |  |
| Feb 2020 | 613 | 6,144 | 7,284 |  | 12,250 | 14,449 |  | 34% |  |
| Mar 2020* | 435 | 5,458 | 6,608 |  | 8,852 | 10,608 |  | 38% |  |
| Apr 2020* | 20 | 3,895 | 4,838 |  | 2,442 | 3,142 |  | 61% |  |
| May 2020* | 124 | 4,077 | 5,191 |  | 2,651 | 3,465 |  | 60% |  |
| Jun 2020 | 892 | 5,149 | 7,034 |  | 3,856 | 5,272 |  | 57% |  |
| Jul 2020 | 1,344 | 5,728 | 8,069 |  | 4,979 | 6,949 |  | 54% |  |
| Aug 2020 | N/A | 4,701 | 6,306 |  | 5,253 | 7,086 |  | 47% |  |
| Sep 2020* | N/A | 5,422 | 7,561 |  | 6,211 | 8,542 |  | 47% |  |
| Oct 2020* | N/A | 5,043 | 6,941 |  | 6,120 | 8,345 |  | 45% |  |
| Nov 2020* | N/A | 3,877 | 5,495 |  | 5,866 | 8,066 |  | 41% |  |
|  |  |  |  |  |  |  |  |  |  |
| *COVID-19 social restrictions (March-May 2020, September-Nov 2020). **No. trial participants/all attendances x 100%. | | | | | | | | | |

**Appendix III. Number of STI tests, diagnoses, and test positivity among trial participants and non-trial attendees to clinics participating in the PrEP Impact trial, January 2019-November 2020**

|  | Trial participants (MSM) | | | |  | Non-trial attendees (MSM) | | | |
| --- | --- | --- | --- | --- | --- | --- | --- | --- | --- |
|  | No. attendees diagnosed | No. STIs diagnosed | No. STI tests | Test positivity  (95% CI)** |  | No. attendees diagnosed | No. STIs diagnosed | No. STI tests | Test positivity  (95% CI)** |
| Jan 2019 | 790 | 904 | 9,813 | 9.2% (8.6-9.8) |  | 991 | 1,106 | 14,780 | 7.5% (7.0-7.9) |
| Feb 2019 | 552 | 635 | 7,514 | 8.5% (7.8-9.1) |  | 810 | 872 | 12,201 | 7.1% (6.7-7.6) |
| Mar 2019 | 577 | 656 | 7,477 | 8.8% (8.1-9.5) |  | 865 | 953 | 13,953 | 6.8% (6.4-7.3) |
| Apr 2019 | 638 | 739 | 8,454 | 8.7% (8.1-9.4) |  | 868 | 958 | 13,289 | 7.2% (6.8-7.7) |
| May 2019 | 722 | 838 | 9,105 | 9.2% (8.6-9.8) |  | 977 | 1,092 | 15,081 | 7.2% (6.8-7.7) |
| Jun 2019 | 619 | 728 | 7,525 | 9.7% (9.0-10.4) |  | 962 | 1,062 | 14,303 | 7.4% (7.0-7.9) |
| Jul 2019 | 718 | 832 | 9,724 | 8.6% (8.0-9.2) |  | 986 | 1,108 | 15,687 | 7.1% (6.7-7.5) |
| Aug 2019 | 770 | 882 | 9,285 | 9.5% (8.9-10.1) |  | 1,035 | 1,137 | 15,602 | 7.3% (6.9-7.7) |
| Sep 2019 | 826 | 969 | 9,685 | 10.0% (9.4-10.7) |  | 1,100 | 1,239 | 15,701 | 7.9% (7.5-8.3) |
| Oct 2019 | 954 | 1,097 | 12,089 | 9.1% (8.5-9.6) |  | 1,140 | 1,261 | 16,022 | 7.9% (7.4-8.3) |
| Nov 2019 | 896 | 1,031 | 11,833 | 8.7% (8.2-9.3) |  | 980 | 1,084 | 15,089 | 7.2% (6.8-7.6) |
| Dec 2019 | 823 | 956 | 10,465 | 9.1% (8.6-9.7) |  | 964 | 1,077 | 13,981 | 7.7% (7.3-8.2) |
| Jan 2020 | 1,112 | 1,320 | 14,541 | 9.1% (8.6-9.6) |  | 1,248 | 1,398 | 18,075 | 7.7% (7.3-8.2) |
| Feb 2020 | 864 | 1,001 | 12,012 | 8.3% (7.8-8.9) |  | 961 | 1,077 | 14,500 | 7.4% (7.0-7.9) |
| Mar 2020* | 729 | 838 | 9,781 | 8.6% (8.0-9.2) |  | 802 | 898 | 9,776 | 9.2% (8.6-9.8) |
| Apr 2020* | 280 | 335 | 2,874 | 11.7% (10.4-13.0) |  | 315 | 367 | 1,945 | 18.9% (17.0-20.9) |
| May 2020* | 342 | 413 | 3,306 | 12.5% (11.3-13.8) |  | 301 | 350 | 2,508 | 14.0% (12.5-15.5) |
| Jun 2020 | 452 | 563 | 5,530 | 10.2% (9.4-11.1) |  | 429 | 490 | 3,917 | 12.5% (11.4-13.7) |
| Jul 2020 | 611 | 720 | 6,980 | 10.3% (9.6-11.1) |  | 531 | 613 | 5,583 | 11.0% (10.1-11.9) |
| Aug 2020 | 625 | 728 | 8,558 | 8.5% (7.9-9.1) |  | 523 | 599 | 5,807 | 10.3% (9.5-11.2) |
| Sep 2020* | 776 | 911 | 10,281 | 8.9% (8.3-9.5) |  | 669 | 765 | 7,676 | 10.0% (9.3-10.7) |
| Oct 2020* | 822 | 959 | 9,299 | 10.3% (9.7-11.0) |  | 671 | 765 | 7,147 | 10.7% (10.0-11.5) |
| Nov 2020* | 673 | 804 | 7,291 | 11.0% (10.3-11.8) |  | 678 | 761 | 6,999 | 10.9% (10.1-11.7) |
|  |  |  |  |  |  |  |  |  |  |
| *COVID-19 social restrictions (March-May 2020, September-Nov 2020). **No. STIs diagnosed/all STI tests x 100%. | | | | | | | | | |

**Appendix IV. Number of HIV tests, diagnoses, and test positivity among trial participants and non-trial attendees to clinics participating in the PrEP Impact trial, January 2019-November 2020**

|  | Trial participants (MSM) | | | | |  | Non-trial attendees (MSM) | | | | |
| --- | --- | --- | --- | --- | --- | --- | --- | --- | --- | --- | --- |
|  | No. attendees | No. attendees HIV tested | No. HIV diagnoses | No. HIV tests | Test positivity (95% CI)** |  | No. attendees | No. attendees HIV tested | No. HIV diagnoses | No. HIV tests | Test positivity (95% CI)** |
| Jan 2019 | 4,488 | 3,320 | 1 | 3,327 | 0.03% (0.00-0.17) |  | 13,359 | 9,589 | 17 | 9,594 | 0.18% (0.10-0.28) |
| Feb 2019 | 3,762 | 2,580 | 0 | 2,585 | 0.00% (0.00-0.14) |  | 11,928 | 8,042 | 25 | 8,043 | 0.31% (0.20-0.46) |
| Mar 2019 | 3,570 | 2,439 | 2 | 2,442 | 0.08% (0.01-0.30) |  | 12,830 | 8,772 | 25 | 8,776 | 0.28% (0.18-0.42) |
| Apr 2019 | 4,040 | 2,866 | 2 | 2,873 | 0.07% (0.01-0.25) |  | 12,267 | 8,326 | 17 | 8,329 | 0.20% (0.12-0.33) |
| May 2019 | 4,794 | 3,269 | 0 | 3,274 | 0.00% (0.00-0.11) |  | 13,007 | 8,983 | 29 | 8,984 | 0.32% (0.22-0.46) |
| Jun 2019 | 4,291 | 2,694 | 1 | 2,695 | 0.04% (0.00-0.21) |  | 12,671 | 8,646 | 16 | 8,649 | 0.19% (0.11-0.30) |
| Jul 2019 | 5,516 | 3,595 | 3 | 3,613 | 0.08% (0.02-0.24) |  | 14,040 | 9,640 | 22 | 9,642 | 0.23% (0.14-0.35) |
| Aug 2019 | 5,805 | 3,461 | 0 | 3,470 | 0.00% (0.00-0.11) |  | 13,736 | 9,436 | 30 | 9,441 | 0.32% (0.21-0.45) |
| Sep 2019 | 5,700 | 3,445 | 0 | 3,454 | 0.00% (0.00-0.11) |  | 13,424 | 9,200 | 25 | 9,200 | 0.27% (0.18-0.40) |
| Oct 2019 | 6,643 | 4,302 | 1 | 4,310 | 0.02% (0.00-0.13) |  | 13,998 | 9,516 | 27 | 9,521 | 0.28% (0.19-0.41) |
| Nov 2019 | 6,301 | 4,274 | 1 | 4,287 | 0.02% (0.00-0.13) |  | 12,957 | 8,745 | 22 | 8,748 | 0.25% (0.16-0.38) |
| Dec 2019 | 5,457 | 3,663 | 2 | 3,665 | 0.05% (0.01-0.20) |  | 11,820 | 7,806 | 18 | 7,806 | 0.23% (0.14-0.36) |
| Jan 2020 | 6,888 | 5,036 | 4 | 5,042 | 0.08% (0.02-0.20) |  | 14,117 | 9,875 | 22 | 9,876 | 0.22% (0.14-0.34) |
| Feb 2020 | 6,144 | 4,274 | 1 | 4,277 | 0.02% (0.00-0.13) |  | 12,250 | 8,070 | 20 | 8,072 | 0.25% (0.15-0.38) |
| Mar 2020* | 5,458 | 3,386 | 0 | 3,392 | 0.00% (0.00-0.11) |  | 8,852 | 5,265 | 20 | 5,266 | 0.38% (0.23-0.59) |
| Apr 2020* | 3,895 | 931 | 1 | 934 | 0.11% (0.00-0.60) |  | 2,442 | 1,024 | 5 | 1,024 | 0.49% (0.16-1.14) |
| May 2020* | 4,077 | 1,191 | 0 | 1,193 | 0.00% (0.00-0.31) |  | 2,651 | 1,363 | 13 | 1,363 | 0.95% (0.51-1.63) |
| Jun 2020 | 5,149 | 2,552 | 3 | 2,564 | 0.12% (0.02-0.34) |  | 3,856 | 2,176 | 14 | 2,176 | 0.64% (0.35-1.08) |
| Jul 2020 | 5,728 | 3,362 | 0 | 3,382 | 0.00% (0.00-0.11) |  | 4,979 | 2,886 | 9 | 2,887 | 0.31% (0.14-0.59) |
| Aug 2020 | 4,701 | 2,831 | 0 | 2,845 | 0.00% (0.00-0.13) |  | 5,253 | 3,057 | 10 | 3,058 | 0.33% (0.16-0.60) |
| Sep 2020* | 5,422 | 3,359 | 3 | 3,386 | 0.09% (0.02-0.26) |  | 6,211 | 3,919 | 7 | 3,924 | 0.18% (0.07-0.37) |
| Oct 2020* | 5,043 | 3,057 | 2 | 3,072 | 0.07% (0.01-0.24) |  | 6,120 | 3,790 | 7 | 3,793 | 0.18% (0.07-0.38) |
| Nov 2020* | 3,877 | 2,416 | 3 | 2,430 | 0.12% (0.03-0.36) |  | 5,866 | 3,580 | 13 | 3,581 | 0.36% (0.19-0.62) |
|  |  |  |  |  |  |  |  |  |  |  |  |
| **COVID-19 social restrictions (March-May 2020, September-Nov 2020). **No. seroconversions/all HIV tests x 100%. | | | | | | | | | | | |

**Appendix V. Number of attendees with PrEP need and percentage of attendees with PrEP need who had need met by the PrEP Impact trial, January 2019-November 2020**

|  | Attendees with PrEP need | | | |  |  |  |  |  |  |  |
| --- | --- | --- | --- | --- | --- | --- | --- | --- | --- | --- | --- |
|  | No. trial attendees** | No. non-trial attendees at risk of HIV acquisition | No. attendees who seroconverted | No. attendees who ever sourced PrEP elsewhere | No. non-trial attendees not at risk of HIV acquisition |  | Total no. SHS attendees | Total no. attendees with PrEP need |  | % of all attendees with PrEP need | % of all attendees with PrEP need met by Impact trial |
| Jan 2019 | 4,487 | 5,722 | 19 | 1,555 | 7,620 |  | 19,403 | 11,783 |  | 61% (60-62) | 38% (37-39) |
| Feb 2019 | 3,762 | 4,790 | 25 | 1,392 | 7,113 |  | 17,082 | 9,969 |  | 58% (57-60) | 38% (37-39) |
| Mar 2019 | 3,568 | 5,267 | 27 | 1,469 | 7,538 |  | 17,869 | 10,331 |  | 58% (57-59) | 35% (33-36) |
| Apr 2019 | 4,038 | 5,068 | 21 | 1,422 | 7,182 |  | 17,731 | 10,549 |  | 59% (58-61) | 38% (37-39) |
| May 2019 | 4,794 | 5,445 | 29 | 1,636 | 7,533 |  | 19,437 | 11,904 |  | 61% (60-62) | 40% (39-41) |
| Jun 2019 | 4,290 | 5,171 | 17 | 1,710 | 7,484 |  | 18,672 | 11,188 |  | 60% (59-61) | 38% (37-40) |
| Jul 2019 | 5,513 | 5,698 | 26 | 1,595 | 8,320 |  | 21,152 | 12,832 |  | 61% (60-62) | 43% (42-44) |
| Aug 2019 | 5,805 | 5,546 | 30 | 1,524 | 8,160 |  | 21,065 | 12,905 |  | 61% (60-62) | 45% (44-46) |
| Sep 2019 | 5,700 | 5,634 | 25 | 1,584 | 7,765 |  | 20,708 | 12,943 |  | 63% (61-64) | 44% (43-45) |
| Oct 2019 | 6,642 | 5,717 | 28 | 1,705 | 8,254 |  | 22,346 | 14,092 |  | 63% (62-64) | 47% (46-48) |
| Nov 2019 | 6,300 | 5,352 | 23 | 1,602 | 7,583 |  | 20,860 | 13,277 |  | 64% (63-65) | 47% (46-49) |
| Dec 2019 | 5,455 | 4,935 | 21 | 1,553 | 6,867 |  | 18,831 | 11,964 |  | 64% (62-65) | 46% (44-47) |
| Jan 2020 | 6,884 | 6,204 | 27 | 1,841 | 7,891 |  | 22,847 | 14,956 |  | 65% (64-67) | 46% (45-47) |
| Feb 2020 | 6,143 | 5,119 | 21 | 1,585 | 7,111 |  | 19,979 | 12,868 |  | 64% (63-66) | 48% (47-49) |
| Mar 2020* | 5,458 | 3,347 | 20 | 1,123 | 5,485 |  | 15,433 | 9,948 |  | 64% (63-66) | 55% (53-56) |
| Apr 2020* | 3,894 | 727 | 7 | 332 | 1,710 |  | 6,670 | 4,960 |  | 74% (72-76) | 79% (76-81) |
| May 2020* | 4,077 | 887 | 13 | 445 | 1,751 |  | 7,173 | 5,422 |  | 76% (74-78) | 75% (73-78) |
| Jun 2020 | 5,146 | 1,442 | 18 | 702 | 2,400 |  | 9,708 | 7,308 |  | 75% (74-77) | 70% (69-72) |
| Jul 2020 | 5,728 | 1,933 | 10 | 1,071 | 3,037 |  | 11,779 | 8,742 |  | 74% (73-76) | 66% (64-67) |
| Aug 2020 | 4,701 | 2,188 | 10 | 1,396 | 3,055 |  | 11,350 | 8,295 |  | 73% (72-75) | 57% (55-58) |
| Sep 2020* | 5,419 | 2,754 | 12 | 1,445 | 3,450 |  | 13,080 | 9,630 |  | 74% (72-75) | 56% (55-58) |
| Oct 2020* | 5,041 | 2,641 | 9 | 1,758 | 3,472 |  | 12,921 | 9,449 |  | 73% (72-75) | 53% (52-55) |
| Nov 2020* | 3,874 | 2,373 | 16 | 1,760 | 3,480 |  | 11,503 | 8,023 |  | 70% (68-71) | 48% (47-50) |
|  |  |  |  |  |  |  |  |  |  |  |  |
| *COVID-19 social restrictions (March-May 2020, September-Nov 2020). **Includes trial participants and non-trial attendees. | | | | | | | | | | | |
